# Our Thanks

**Published:** 1872-03

**Authors:** 


### OUR THANKS.

We sincerely thank those gentlemen who have sent us communications. We will publish as early as possible. We trust our friends everywhere will send us articles. We also thank our friends for the number of pamphlets which have been forwarded us from various sections of the country. We hope authors of books, pamphlets, addresses, etc., will forward their productions to us for notice, as a means of bringing them before our readers, as the libraries of many of our professional brethren were lost during the late war.

We thank those of our friends who have made suggestions and given us advice on certain points in the conduct of our journal. By all means, keep us posted. We wish to make the COMPANION worthy of the profession, and will labor to that end.

Letters requiring answers will be replied to as early as possible, under the pressure of our large and increasing correspondence.

